# Status of Hospital Domestic Service

**Published:** 1920-08-28

**Authors:** Florence A. Crombie


*To the Editor of* The Hospital.

### Status of Hospital Domestic Service.

*To the Editor of* The Hospital.

Sir,—Would it not be a work of philanthropy to secure young girls leaving "Homes" and "Orphanages" at about sixteen years of age as ward-maids in our hospitals? They are used to "routine" and trained in domestic work—*i.e.*, in cleaning their own dormitories, etc.—and ward work would in a manner correspond. Besides their seeming fitness, the life would be good for them morally. Matrons and nurses live for their patients, and may not these girls catch the same spirit and the same ideals of living? There is much in institutional life to humanise and "educate" the girl brought up in a "Home," and being of impressionable age she would also, with youthful eagerness, rejoice to be useful in times of greater stress. We want willing workers in our institutions, and who more willing in every way than the young? To pay these girls £12 a year and provide uniform would be comparatively cheap labour to the institution, but the institution must see to it that these girls have eight hours only on duty and that there are comfortable quarters for their leisure. Would it not be a good arrangement to provide a superintendent for these young maids on the threshold of life, to instil sound principles while helping them to be happy and joining in their recreations? This would be cheaper than the present-day system.—Yours faithfully,

Florence A. Crombie.

